# Book Reviews

**Published:** 1819-03

**Authors:** 


### CRITICAL ANALYSIS

### OF RECENT PUBLICATIONS,

IN THE

### DIFFERENT BRANCHES OF MEDICINE, SURGERY, &c.

*Medico-Chirurgical Transactions, published by the Medical and Chirurgical Society of London.* Vol. IX. Part I.—Longman and Co. 1818.

*(Continued from p. 156.)*

IX.—*Experiments and Observations on the Union of Fractured Bones;* by JOHN HOWSHIP, Esq.

IN the detail of these experiments and observations we discern much minuteness of research, with correctness of exposition, highly valuable to the physiologist; but to which a curtailed abstract, unaided by delineations, would fail to do justice.

X.—*Brief Notice presented to the Medico-Chirurgical Society, with the original Obstetric Instruments of the Chamberlins;* by H. H. CARWARDINE, Esq.

The object of this notice is to establish the authenticity of the obstetric instruments deposited by Mr. Carwardine in the possession of the Medico-Chirurgical Society, as those which were the invention, and had been the property, of the Chamberlins. These instruments were discovered, two or three years ago, in a secret receptacle under the floor of a closet in Woodham Mortimer Hall, near Maldon, in Essex; which estate was purchased some time previous to 1683, by Dr. Peter Chamberlin, and remained in the possession of his family till about 1715. The instruments claim an interest from their former possessors, and bear, according to Mr. Carwardine, intrinsic marks of originality of invention.

XI.—*Case of Aneurism in the Arm, cured by tying the Subclavian Artery;* by Dr. POST, of New York. Communicated by Mr. Cooper.

A record of a successful operation on the subclavian artery, for the cure of aneurism extending into the axilla.

XII.—*A singular Case of Expulsion of a blighted Fœtus and Placenta at Seven Months, a living Child still remaining to the full Period of Utero-Gestation;* by JOHN CHAPMAN, Esq. member of the Royal College of Surgeons, and Surgeon in Windsor. Communicated by Dr. BAILLIE.

This very extraordinary deviation from the ordinary course of nature occurred in the practice of Mr. Chapman, in the month of October, 1816. His patient, Mrs. R. of Clewer, about the seventh month of pregnancy, was seized with hæmorrhage and pains, which led to the expulsion of a fœtus, about the usual size of those between three and four months. It was enveloped in membranes apparently unopened, but devoid of liquor amnii, and accompanied by its placenta. After this, the pains and hæmorrhage subsided; and, though it was observed at the time that another fœtus alive remained in the uterus, there was no recurrence of either. She was kept quiet, and completed the full period of utero-gestation, when a full-grown living female child was born. The preparation of the blighted fœtus is deposited with the Society.

XIII.—*Some Observations on one Species of Nævus Maternus; with the Case of an Infant where the Carotid Artery was tied;* by JAMES WARDROP, Esq. F.R.S. Ed.

The species of nævus maternus which forms the subject of Mr. Wardrop's present paper, is the one which is situated under the skin, forming in the cellular structure betwixt the skin and the superficial muscles; and which, with propriety, he proposes to distinguish by the appellation of the subcutaneous nævus. This tumor is congenital, and not confined to any part of the body, though most frequently found about the face. Its limits are distinguishable by the touch, its form flattened, and at first it is freely moveable betwixt the integuments and muscles. The superjacent skin retains its natural colour till the tumor becomes prominent, when the large vessels shining through give more or less of a purple cast. When small, it has a doughy feel, and seems to have little sensibility; may be diminished in volume by squeezing it, and it enlarges when the child cries. This nævus has no distinct pulsation, but an universal throbbing perceptible on squeezing it. When the tumor is large, it has blood-vessels of a large size passing into it, rendering its removal by the knife extremely dangerous.

Sometimes the tumor is small, and continues stationary; sometimes it diminishes spontaneously; at other times it slowly increases, not putting on a serious aspect till late in

1

life. Sometimes, particularly when the tumor is small, ulceration takes place in the covering skin, and this, with part of the tumor, sloughs. The ulcerated surface finally heals, and, though some portion of the tumor may remain, its progress is arrested. Occasionally its appearance at birth is formidable; the skin, distended and discoloured, gives way, and a fatal hæmorrhage ensues. On examination of one of these tumors, which Mr. Wardrop had removed, thinking it the only means of saving the child's life, the skin having given way, and hæmorrhage to an alarming extent having occurred,—the structure was spongy, composed of cells and canals, into which the large vessels supplying the mass opened. The internal surface of these cavities was polished and shining; in some places bearing a resemblance to the cavities of the heart, having something like the cordæ tendineæ.

From the inapplicability of cold and pressure in some instances, and the danger of hæmorrhage in the operation, the treatment of this species of nævus is seldom very successful. In a case where the tumor occupied the greater part of the side of the face and head, and ulceration had commenced, Mr. Wardrop was induced to tie the carotid artery, as the most probable chance of checking the supply to the morbid mass. The effect of the operation on the tumor was promising, and, though under very unfavourable circumstances of debility, gave hopes of success: the child, however, sunk on the fourteenth day, exhausted by the irritation of an extensive ulcerative process, and by previous depletion.

Mr. Wardrop concludes his observations on the nævus subcutaneus by some valuable practical precepts on the treatment, as suggested to him by his own experience in these cases. He says, " Tumors of this description may be removed by the knife, by ulceration, by absorption, by tying the vascular trunks supplying them, and by ligature: these different means being employed singly or combined, as may appear best adapted to the individual case."

When the tumor is small, or even of moderate size, it may be removed with safety from any part of the body by the knife, using especial caution in such cases not to cut into the tumor. By making the incisions beyond the bounds of the tumor, the hæmorrhage is less considerable, and ceases after the extirpation of the diseased mass. When the ulcerative process seems preferable, it may be begun by destroying a central portion of the skin by lunar caustic or kali. Should the ulceration advance too rapidly, it may be powerfully controled by the application of the balsam of Peru to the surface of the sore. Where the tumor is so large as to render either of these modes of extirpation hazardous, it seems advisable, where it is practicable, to tie the trunks of the larger nutrient vessels; whereby diminution of bulk will be obtained, and the risk of hæmorrhage lessened, whether recourse be had subsequently to excision or the process of ulceration. Mr. Wardrop has not used the ligature, but he says that it has been once adopted by Mr. White with success.

XIV.—Mr. Wardrop has subjoined to his paper *a Case of Aneurism by Anastomosis*, which was successfully treated by Mr. Lawrence. The disease occupied the first phalanx of the ring finger on the right hand, the chief swelling being on the palmar surface and ulnar side of the finger. The tumor had pulsation, which was stopped by pressing on the radial and ulnar arteries. These trunks were tied; but after some time the state of the disease was just the same as before. Compression was ineffectual. Mr. Lawrence made a circular incision close to the palm, through all the soft parts, excepting the flexor tendons with their theca, and the extensor tendon. The digital artery, the pulsation of which had been very evident in the palm, was fully equal in size to the radial or ulnar of an adult, and was the principal nutrient vessel of the disease. After tying this and the opposite one, it became necessary to place ligatures on the other orifices of these vessels. The edges of the incision were brought together by four sutures, but the parts could not be very satisfactorily united, from the swelling of the part beyond the cut. The wound healed slowly; the swelling subsided, but did not entirely disappear; and the integuments recovered their natural colour. The pulsation and pain were put an end to. The natural powers of the part were so far recovered as to enable the patient to work some time at her needle, and to use the arm for most purposes.

XV.—*Notes of a Case of Mercurial Erethism;* by T. Bateman, M.D. F.R.S. &c.

We have in this communication a report of singular interest and value. The serious character of an affection which is sometimes induced by the very remedy on which we are reposing confidence for the removal of some other morbid condition, gives an importance to every occasion of extending our acquaintance with the disease; but the case immediately before us presents a vastly-augmented interest, from its having occurred in the person of one so eminently qualified to mark the minutest agency of the poison on his system, as the learned and scientific narrator of the case himself.

A course of mercurial inunction was entered upon, under the sanction of the first medical authority, with a view to relieve an amaurosis of the right eye, which had come on during the preceding summer, in connexion with a considerable derangement of the chylopoïetic functions, and was commenced after a residence of three months at Brighton; during which time these functions and the general health had been materially improved.

A drachm of mercurial ointment was nightly rubbed in, and in about a week tenderness of the gums, with nightly febricula, were induced. The first notice of the deleterious action of the medicine upon the system appears to have shown itself in an attack of violent and irregular beating of the heart, which came on about four in the morning of the ninth day, and did not yield to laudanum and stimulants, but suddenly subsided about 1, P.M. A feverish state ensuing with much griping, and on the following day the irregularity of the circulation recurring, the mercury was omitted one night. Palpitation having gone off during sleep, and the feelings being more comfortable during the subsequent day, the friction was repeated at night; but, towards morning, the heart's action became again disturbed. This disturbance continuing, it was determined, in consultation, to desist altogether from the use of mercury for the present. The irregular action of the heart continued without remission for some days, with diminution of strength, though not so much as to prevent the patient from quitting the house. The stomach began to fail in its functions; flatus being generated, and cough induced, which, when violent, brought on retching, but not vomiting. The stimulus of spiced wine became necessary to subdue these symptoms. On the seventeenth day, the languor was considerably increased, from his having the preceding night been feverish and sleepless. From this time he was unable to leave the house. The languor, debility, and cough, rapidly increased, accompanied by a distressing sense of constriction across the region of the diaphragm: this, with the continuing perturbation of the heart, made it necessary to remain in nearly an upright posture, even during the night. It was necessary to augment the allowance of spiced wine to a bottle in the twenty-four hours. The nights were passed almost without sleep, from the disturbed circulation, the constant cough, and painful distension of the stomach by flatulence. In the night of the twenty-second day, being propped up on a couch, the action of the heart became once more regular, and the succeeding day was passed with less suffering. On the following night, resuming the same position, the patient fell into a tranquil sleep for about an hour, when he awoke with a start, and with a momentary confusion of thought, calling out in apparent distress for the admission of fresh air. On the return of consciousness, the irregular action of the heart was found to have recommenced. The tranquillity of the preceding period had been attributed to extract of hyosciamus, which was taken now without effect. Tincture of hop, which was substituted, produced only drowsiness. To obviate the increased languor and debility, supplies of food and cordials were repeated more frequently, and continued during the night. Again, during the night, a disturbed sleep of only a quarter of an hour was interrupted by a start and confusion, and a still more importunate demand for fresh air, which it became necessary to meet by opening the windows and doors, and drawing the couch into the current. Two subsequent nights were passed somewhat similarly; and on the following the interruption of a short sleep was accompanied by a sensation of sinking, which was felt as that of approaching dissolution. Fresh air was anxiously demanded, and undiluted brandy urgently called for: this was taken to the extent of three glasses in the course of five minutes, without much relief. Ammonia and æther were substituted, one or other of which was repeated every ten minutes for about two hours; when the faintness rapidly declined, especially after a very copious discharge of limpid urine. During this paroxysm, notwithstanding the total depression of muscular power, and the feeling of sinking to immediate death, the mental powers remained clear, the extremities warm, and the pulse (though extremely irregular, and not according exactly in its beats with the contractions of the heart,) was felt in all the extremities. A second and similar paroxysm occurred in about two hours, with still greater depression, which yielded again to stimulants, leaving, however, greater languor. It appeared that the action of the heart and arteries, extremely feeble and irregular whilst awake, was still more enfeebled during sleep, so as to be in fact almost suspended, and thus to occasion those alarming faintings and sinkings. It, therefore, became necessary to interrupt the sleep at the expiration of two minutes, by which time, or even sooner, the sinking of the pulse and countenance indicated the approaching languor. This interruption was necessarily continued for three weeks or more, the periods of permitted sleep being gradually extended. In this state of debility, the powers of the stomach seemed rapidly to fail, it being incapable of receiving the accustomed solid animal diet. A grain of cayenne pepper was taken every hour for eleven or twelve hours. Extreme distress from flatulence remained, and the cough and faintings still continued to harass. Stimulants relieved, but the most marked advantage was derived from musk: this drug seemed to diffuse its stimulating effects through every part of the system. Cinchona was now used in several forms. The distressing symptoms recurred at intervals; and did not seem to abate until a change of diet was made, relinquishing the preparations of animal and vegetable food for asses' milk taken alternately with that of cows: these were, by slow degrees, changed for vegetable, and at length solid animal, diet. Each change, however, was attended by oppression and flatulence. With the quality of the food was gradually extended the periods of abstinence. Food was for some time required during the night; and the necessity of maintaining an erect posture continued during several weeks. A slow subsidence in the disturbance of the heart's functions took place, and regularity of action and muscular strength were by degrees re-established.

The interest of this case will, we trust, be such as to fully warrant this somewhat extended detail.

XVI.—*On the Effect of Nitrate of Silver on the Complexion;* by Dr. Badeley, of Chelmsford. Communicated by Sir Henry Halford, Bart.

In this case, the nitrate of silver was successfully administered for epilepsy, in doses of a grain to a grain and a half three times a-day, continued during a year and a half. The change in the colour of the skin occurred after the discontinuance of the medicine, and at the expiration of nearly two years retained its leaden colour. The bosom was somewhat darker than the face, with a purple hue. The roof of the mouth, inside of the cheeks, and back part of the tongue, dark; the tunica schlerotica, much discoloured. Blisters on the discoloured surface rose white. The patient had been free from any symptom of epilepsy during two years and a half.

XVII.—*Case of an extensive Wound from the Bite of a Shark;* by Dr. Kennedy. Communicated by Sir James Macgrigor.

A poor diver, in India, had received from the jaws of a shark a frightful laceration of the muscles of the abdomen, pelvis, and thigh. The cavity of the abdomen was extensively laid open, and the exterior of the pelvis and upper part of the thigh dreadfully mangled. By the skilful treatment and solicitous attention of his surgeon, aided doubtless by a healthful temperament, this alarmingly-extensive injury was repaired, and the poor sufferer restored to his sphere of usefulness in society.

XVIII.—*A Report of the principal Diseases which occurred among the Gentlemen Cadets in the Royal Military College at Great Marlow, Bucks, and Sandhurst, Berkshire, during a period of Seven Years; viz.—from 3d of September,* 1809, *to 2d of September,* 1816. By N. Bruce, Esq. A.M. Surgeon to the Forces, and to the Royal Military College.

In this collective view of the course and character of disease manifesting itself in an extensive assemblage of individuals, placed under almost similar circumstanees of age, diet, and discipline, the reader of the Transactions will follow, with interest, the historical sketch, the medical comment, and the therapeutic doctrine, of the able reporter; but the extent to which our notice of the present volume has already gone warns us from entering on the particulars of a paper, which, from its nature, scarcely admits of abstract.

---

*A Manual of Practical Anatomy, for the use of Students engaged in Dissections;* by Edward Stanley, Assistant-Surgeon and Demonstrator of Anatomy at St. Bartholomew's Hospital.—12mo. pp. 439. Anderson and Chase, London, 1818.

"This work," the author observes, in a Preface, "is strictly for the dissecting-room. Its objects are to describe accurately the several parts of the human body, and to give practical directions for the most convenient method of dissecting them. It has no other plan but to arrange the several regions of the body in the order most convenient for their examination, and to describe each successively according to its natural situation. Let the reader, who turns from the perusal of systematic works to this Manual, recollect that it is intended only for practical beginners in the science, to facilitate their first difficulties, and to furnish them with the sort of instruction which they especially require."

It is only necessary to remark, that Mr. Stanley has effected the objects he has proposed, and in a manner which, for precision and clearness, can hardly be excelled. We would apply part of the motto chosen by the author in a sense somewhat different from that in which his modesty has led him to employ it:—"*methodus sola* ARTIFICEM

*ostendit;*" and think that this work is not adapted for students alone, but that it may also be consulted with much advantage by those surgeons whose anatomical knowledge may have "grown rusty," previously to the performance of many operations.

The author has confined his observations solely to *anatomical* description; the uses of parts constituting another branch of knowledge, which should be subsequently acquired, and in *retirement* rather than in the dissecting-room,—as it is there alone that works written according to the ideas of Bordeu, will be productive of their full influence on the mind of the student.

---

*An Enquiry into the Influence of Situation on Pulmonary Consumption, and on the Duration of Life. Illustrated by Statistical Reports.* By JOHN G. MANSFORD, Member of the Royal College of Surgeons of London.—1818.

No enquiry which has for its object the means of circumscribing within narrower bounds so widely-spread, and so generally-fatal, a malady, as phthisis pulmonalis, can be met with indifference. Hitherto this disease seems to mock the unavailing opposition of the healing art; and he who shall afford a surer check to its merciless career, will be a benefactor of his race. The influence of climate in these cases appears to have been long and extensively acknowledged, if we regard the universal manumission which this unfortunate class of sufferers has been accustomed to receive: after demonstrating in almost every variety of locality the futility of medicine, and the baffled skill of their physician, then are they dismissed "to try a change of air." Little, perhaps, as may many of the selected places of retreat fulfil the delusive anticipations of their devoted visitants, it is, we are sure, quite reasonable to build a hope, nay to frame an expectation, on the auxiliary influence of local situation and climate in controling pulmonary disease. But this aid should be courted in our earliest notice of the affection, not reserved as a dernier resort, when all our other means shall have been unavailingly exhausted. The circumstance that has, perhaps, almost exclusively operated in the selection of a residence for the consumptive patient, has been temperature; and this certainly is not one of the least important, for it is very manifest how noxious must be abrupt and violent alternations of external temperature on a frame so little fitted to resist the impression of such changes. But temperature is not the only influential agent, adverse or salutary, which claims our attention in the choice of locality for the treatment of phthisis pulmonalis. Mr. Mansford, in the present essay, has brought before our notice facts that imperiously direct our views in the consideration of this disease to the agency of atmospheric pressure. This circumstance, in its participation in the production of deranged actions in the animal economy, has ere now attracted the incidental notice of pathologists; but we believe that it has not before excited the full investigation and specific application which has been devoted to it by the author of the present treatise.

To establish the position that mere change of atmospheric pressure is decisive in its effects on the ordinary actions of the animal frame, Mr. Mansford has very properly recourse to those well-attested instances of obvious derangement occurring immediately on changes of pressure, at once rapid and considerable in extent. He says—

"The sensations experienced by æronauts, and by those who have ascended high mountains, may throw some light on this subject. These sensations have been described as occasionally resembling those of intoxication. At others, great lightness and agility have been experienced, but soon terminating in weariness; hæmorrhages from the nose and lungs; sickness; difficulty of breathing, and hurried respiration on the slightest exertion; a quickened pulse; painful distension of the eyes and ears, and palpitation of the heart. Dr. Halley relates of himself, that he experienced very painful feelings on ascending from a great depth in a diving-bell; the circulation not being immediately able to accommodate itself to the sudden removal of so great additional pressure."

These are marked effects, readily cognizable and immediately deducible from an obvious cause. They are, it is allowed, extreme cases, and such as differ widely from the effects to be expected from the more gradual and more moderate transitions made by change of station on the common habitable surface of the earth. But similar effects will still be produced, modified only in proportion to the intensity of their causes; and, admitting them in the extremes, we cannot deny them in their subordinate gradations. The author shows, too, that ordinary variations of elevation in places of accustomed habitation influence, in a discernible manner, some of the leading phenomena of the living system. Thus he has found, by repeated experiment, that an elevation of five hundred feet, which is not an unusual difference in situations not remote from each other, has caused an acceleration in the pulse of five or six beats in a minute; and that any motion of the body has produced a greater corresponding increase of pulsations in the high situation than in the lower one. If this (as Mr. M. justly remarks) be the effect produced on a system in full health, how much more considerable may it not be expected to be found when disease has weakened the controling power of life, when the balance of the system is disturbed, and the delicate tissue of the lungs has become enfeebled or disorganized. An elevation of five hundred feet is considered to diminish the average of atmospheric pressure somewhat more than a sixtieth part, or nearly six hundred pounds on the surface of the human body:

—" and, although this reduction of pressure is not felt, it cannot be doubted but that the removal of so large a degree of resistance must give greater freedom of action to the main spring of the circulation, as well as a greater power of distention to the vessels themselves, especially, as was before remarked, to the superficial vessels: both of which causes, like all others, will operate most powerfully upon a diseased part.

" In such a state of the pulmonary structure, the action of its vessels, from their greater irritability, and their exposed and unprotected situation, will be increased in a ratio greatly exceeding that of the other vessels of the body; and, where a healthy person finds no change in his feelings, a patient thus circumstanced will experience oppression at the chest and difficulty of breathing, from the greater influx of blood into the pulmonary vessels."

Mr. Mansford refers to observed effects on asthmatic and consumptive patients from change of atmospheric pressure, marked by a considerable variation of elevation in the barometric column. On two succeeding days, when an unusual rise in the mercury had taken place, he found in three patients, whom he made the subject of careful examination, a diminution in the frequency of the pulse of from eight to sixteen beats, with a very perceptible alleviation of distressing symptoms, betwixt a change of pressure marked by 28·3 and 29·1 of the barometer. The improvement was uniformly apparent in all the three. The result of this experiment was in a great measure undisturbed by simultaneous changes of temperature and moisture, which leaves it certainly in a more satisfactory state. Polypous tumors, it is observed, undergo a remarkable alteration of bulk during these changes of pressure of the atmosphere. Facts such as these leave little room for doubt that the general actions of a diseased or debilitated system are susceptible of a powerful influence from variations of pressure on its surface.

Assuming it, then, as a principle, that the progress of pulmonic disease, more especially phthisis, will be sensibly affected by permanent states of relative pressure, the author proceeds to investigate, on statistical reports, how far his reasoning is borne out by the proportional mortality of consumptive patients in situations which differ considerably in elevation: and here there is an extraordinary coincidence, which gives a high value to Mr. Mansford's opinion, and almost carries conviction with his argument.

The county of Somerset, in which the author has chiefly pursued these enquiries, presents a variety of surface admirably fitted for affording a solution of the question which he had instituted. It distributes itself into two extensive ranges,—one of low and flat land, little elevated above the level of the sea; the other a hilly tract, the general surface of which rises to a level of considerable height. From the several towns in these two districts, Mr. Mansford has collected reports of the number of deaths which have occurred in the space of one year from phthisis pulmonalis, with the proportion which these bear to the whole population of each place. The result of this enquiry shows a remarkable affinity betwixt the points of elevation and the mortality from consumption. Local causes, independent of atmospheric pressure, doubtless have their influence in modifying the general prevalence of the disease in certain situations; but, in the range submitted to our immediate notice, the accordance is striking betwixt the increased frequency of phthisis and the more elevated situation of the local point. The period of observation is too limited, and the range of investigation may perhaps be too circumscribed, to warrant any general conclusions; but the results here are too striking not to press the subject upon more extended notice. In the higher range of country, the mean elevation of which is estimated at about 674 feet, the average mortality from consumption appears to be 4 07 to 1000 of the living; whilst in the low plain, which does not rise on the average above 45 feet from the level of the sea, the proportion of deaths from the same cause is not more than 1.52 to 1000 living. This is a difference so great as to make it an object of much interest to ascertain whether a more diffused examination of similar localities will furnish corresponding effects of situation.

"There is one circumstance of a practical nature arising out of this part of the present enquiry of vast importance. The relative prevalence of consumption, in high and low situations, although leading to an inference exceedingly satisfactory, does not afford a true measure of the effect which we may expect will be produced, by a removal to the one situation, of a person who has resided in the other. Where there is an undoubted predisposition to the disease, or where its admonitory symptoms may have already shown themselves, a removal to a lower situation, when practicable, may avert it in the one case, and suspend its progress in the other: while, on the other hand, quitting the accustomed situation to reside in a higher one may call the disease into immediate action where latent, and give it new force where it has already commenced. The natural powers of the constitution, in the situation to which it has been habituated, may be able to maintain for a time the struggle with its deadly foe, and to postpone the visible advances of disease: but, if a powerful auxiliary be abandoned by removing to a more elevated scite, the disease, once set free from constitutional control, will run its course with fatal rapidity. A medical friend informs me, that two members of his own family sunk rapidly under pulmonary consumption, after quitting low situations to reside in elevated ones. A similar fate attended a medical gentleman, who practised some years ago at Wells, who fell a prey to the same disease shortly after leaving that city to practise in an elevated part of Gloucestershire. The universal fatality attending this disease in the numerous cases which are reported to have resorted to Richmond in Yorkshire, will also illustrate this part of my subject; and I think it will not be asserting too much to say, that, to remove to situations higher than that which had been previously inhabited, in any stage of pulmonary consumption, is to run into the very jaws of death."

Pursuing the enquiry into the comparative prevalence of the disease in low situations, Mr. Mansford finds that the immediate vicinity of the sea has some circumstance connected with it which countervails the advantage of a denser medium, and raises the proportion of mortality from pulmonic disease above what it appears to be in low inland situations. Places of this latter description, which enjoy a tolerably equable and somewhat warmer temperature, with a seclusion from the keen blasts of the north and east, are the situations to be selected for those who are actually suffering under, or who may have, a predisposition to pulmonary consumption. On reviewing various points in our own island, Mr. W. finds none which presents so many requisites for the residence of patients of this class as the low ground which extends southward from the Mendip Hills; of which he thus depicts the leading features:—

"Its geographical position is in the south-western part of the island The shelter afforded by the range of hills towards the north, and the lowness of its level, while spots may be chosen just sufficiently raised above the marshy lands to escape the prejudicial and chilling influence of concentrated moisture, without being so high as to defeat the object in view, point it out as one of the most eligible. To these advantages of a physical nature may be added others of a more obvious and inviting character. The varied and romantic scenery of the neighbourhood does not fail to charm those who possess a relish for the beauties of nature; while the tastes and habits of individuals may be gratified in the society of a city, or the seclusion of a village."

On the influence of temperature, which has hitherto been almost solely regarded in the choice of situation, the author shows, from collected reports, that the climate of the south, which is commonly hailed as the most auspicious to the consumptive patient, is not, even in those spots which have been resorted to in preference, so exempt from the disease as many situations in the more rugged climate of the north. But this difference Mr. M. ascribes to relative differences in altitude. This subject is of importance, and will, we trust, receive appropriate consideration from the profession generally; so that we may be put in possession of more numerous data on which to found some definite conclusions. If it can be unequivocally shown that a diminution of superficial pressure permanently abates, however trivially, the control over a disposition to morbidly-increased excitement; and that the actions of the system do not, in a little time, adapt themselves to the altered pressure, so as to regain their original rate; then shall we be assured that we have exchanged a vague and blind reliance on the fortuitous operation of unknown causes, for a rational and definite principle of action: a power hardly perceptible in its momentary action, incessantly applied, effects mighty results.

The second part of Mr. Mansford's essay is devoted to the consideration of the influence of atmospheric pressure on the general duration of life. In the former part, it has been shown to be powerfully operative in repressing the morbidly increased activity of the vital powers. At a period when the balance may be supposed to be nearly equal betwixt the moving powers and the resistance, to lessen the latter will be to give an increased force to the former. This will be the case where the resistance from superficial pressure is diminished; and thus will the actions of the machine be carried on with less expenditure of motive power. But statistical reports agree in showing the greater average longevity of the inhabitants of elevated situations. This may not altogether arise from the diminished pressure of a rarer medium; but this circumstance cannot fail to exert, perhaps, the largest share of influence; and it is a fair inference, therefore, to deduce, that, under a resistance diminished by an abatement of external pressure on the surface, the healthful actions of the animal body are carried on with less exhaustion of the vital powers.

But it is not necessary, in this place, to follow the author through all the facts that go to substantiate his opinions. They will not escape the scrutiny of the medical philosopher, who, we are persuaded, will hold himself indebted to Mr. Mansford for the novel and rational views which he has presented in this interesting essay.

---

*General Views relating to the Stomach, its Fabric and Functions;* by T. C. Speer, M.D. Physician in Bath.

In this little tract the author has brought together the principal facts and doctrines respecting the structure and functions of one of the most important viscera of the animal economy. Proportionate to the importance of its office in the system, is the urgency of the claim which the stomach prefers on the attention of the physiologist and of the physician. So essential to the well-being of the general system is the healthy condition of this organ, and so widely and rapidly are propagated the effects of its functional derangements, that we are necessarily led to regard it, more than any other point in the human frame, as the source of health or as the focus of disease. The physiologist marks, in the function of this viscus, the first link in the connected chain of actions subservient to animal vitality: the pathologist, viewing the important and diffusive influence of the stomach, and its peculiar exposure to the agency of noxious causes, in the great majority of instances, has his attention directed hither, as to the centre of constitutional disturbance. Viewing the subject in these bearings, we are compelled to admit that it is one with which we cannot be too intimately acquainted; and, therefore, the present volume comes with a powerful commendation to notice, and, in many points of its execution, merits our regard; yet we must confess that the impression left by a careful perusal of it was, that the views which it gives of the subject are somewhat too general to contribute importantly to that minuteness of information for which the professional reader is in the habit of searching; but that it is rather calculated to satisfy the enquiries of the student of general philosophy. We do not consider that it will be unprofitably perused by the medical reader, but we think that it will come to him rather as a recapitulation of anteriorly-gathered knowledge, than as an extension of his acquaintance with the subject; and, as an elementary introduction to the junior reader, it wants something of precision and fullness of description, and clear exposition of ascertained facts demonstrative of the functions of the organ,

*Practical Observations on the Nature and Treatment of Marasmus, and of those Disorders allied to it which may be strictly denominated Bilious.* By JOSEPH AYRE, M.D. Member of the Royal Medical Society of Edinburgh; one of the Physicians to the General Infirmary at Hull; senior Physician to the Hull and Sculcoates Dispensary; and Physician in ordinary to the Lying-in Charity, at Hull.—8vo. pp. 256. London, 1818. Baldwin and Co.

UNDER the term Marasmus, Dr. Ayre proposes to consider certain morbid conditions, which, according to the predominance of particular symptoms, have, by different authors, been designated under various appellations as distinct diseases. The diseased state originates in disordered function of the digestive system, and leads to that defective nutrition of the body which produces the obvious external character which is here adopted as distinctive of the morbid affection. The author considers the marasmus of children, and the disorder of adults commonly termed bilious, as disease of the same origin and nature; modified, perhaps, by differences in the system dependent on differences of age and constitution; and he, therefore, uses these terms indiscriminately, and comprehends under them every form and variety of the complaint. The disease is distinguishable into two stages,—an acute and a chronic one; their separate existence is often overlooked, though tolerably well defined by different symptoms. In the chronic form, there is a morbidly-craving appetite, without much thirst or fever; whilst, in the acute, there is a very marked loss, or an absolute extinction, of appetite, with a considerable degree of both thirst and fever.

Dr. Ayre proceeds to describe, with great perspicuity, the symptoms which characterize the progress of marasmus in infancy, in childhood, and in the adult age. They are all indicative of deranged function in some of the chylopoïetic organs; but we cannot here detail them. He remarks its frequent resemblance, in children, to the irritation of difficult dentition or of worms in the intestinal canal, to tabes mesenterica, and to hydrocephalus internus. It is often confounded with dyspepsia, and the mild hysteria of women, and hypochondriasis of men. In the youth of one sex, it passes under the name of chlorosis; and, in the other, it is frequently denominated a chronic weakness. But the most important diseases which it resembles, and from which it is of most consequence to distinguish it, are the anasarca of debility, phthisis pulmonalis, and the organic and inflammatory affections of the liver.

It is only necessary to reflect on the importance of the perfect action of the digestive apparatus to the maintenance of a healthy condition of the entire system, to be convinced of the multiplied variety of secondary disturbances which may result from a derangement of the primary action in the series of animal functions. The necessary consequence of a first imperfect action is the imperfection of a second, which stands in the relation to it of effect. But, independently of such concatenation of disorder, which may be shown to be of physical necessity, there exists in the economy of animate beings another pervading medium, through which disturbance is propagated remotely through the system: strike upon the first link of the chain of sympathies, and vibration runs through its whole extension. Hence the varied course which derangement of function may pursue; and hence the difference of character which disease may ultimately assume. From this source, too, no doubt, has emanated the endless enumeration of diseases which swell the pages of nosologists. We would not be thought to imply that all disease is of one origin, and that this origin is to be sought in chylopoïetic disorder; the complex machinery of animal existence is amenable to the influence of many other extrinsic agencies than those which minister to its nutrition, and all of them may prove the cause of some irregularity of movement. Yet we are persuaded that all of those inlets to disorder, conjoined, form but a trivial proportion to that great avenue, the *iter ad ventriculum;* and we also feel a conviction that medicine has often and long been engaged, and too often worsted, in the contest with affections as of an idiopathic and independent character, which were the secondary, or perhaps more remote, result of derangement introduced into the incipient functions of alimentation.

Dr. Ayre shows that many of the symptoms assumed as characteristic of original diseases above alluded to, are but the product of disordered digestion; and that they are removable by the means which correct the primary affection. He does not combat the existence of such several diseases; but he makes it apparent that their idiopathic nature is frequently very liable to misconception. The distinctions which the author draws betwixt the fictitious diseases and their prototypes are clear and satisfactory. This diagnostic part of the work is full of interest.

Diseased function may be frequently, and for a long continuance, present, when no lesion of organic structure shall be afterwards discoverable, and the symptoms may be such as to induce a belief that organic disease exists; whence, as the author judiciously remarks, highly pernicious practice may be adopted. Minute and extensive examination has convinced Dr. Ayre that defective action is much more rarely to be ascribed to change of structure in an organ than it is common to suppose. His opinion on this subject, with regard to that important viscus—the liver, does not coincide with that of some of the profession, whose opportunities of judging have been very extensive. Some valuable observations occur in the consideration of this part of the subject.

"A considerable number of the symptoms of the bilious affection are produced by the operation of that law of the animal economy which we term sympathy; for, beside the general and local disturbance which is observed to arise directly from the disordered actions of the liver and the other chylopoïetic viscera, there are several important affections produced through the sympathetic connexion subsisting between these organs and different parts of the system, whereby an irritation, present in the former, is communicated to parts of the body with which they have no local nor apparent relation. Of the effects resulting from the agency of this law, there may be said to be several kinds.

"The first we may notice is an increased action of the serous and mucous membranes, whereby a larger secretion of their proper fluids, or a morbid change in them, is produced."

Of the sympathetic establishment of diseased action in these structures, several instances are adduced; leading, in some cases, to partial or general anasarca; in others, to accumulation of serous fluid in particular cavities, and lymphatic depositions in other parts; and, in mucous membranes, to increased action, to purulent secretion, and even to ulcerative process. From the operation of this law, the author deduces the formation of topical, and sometimes formidable, disease, in remote, and frequently important, organs. Hence a frequent cause of difficulty in discriminating betwixt symptomatic and idiopathic disease. On this point the author enlarges to some extent; particularly in treating on the sympathetic affection of the mucous membrane lining the cavities of the respiratory organs. It is of great importance to discriminate betwixt irritation of this nature and the cough of phthisis.

Having treated at some length on the several affections considered as sympathetic, and shown in various instances, occurring in his own practice, their co-existence with biliary derangement, Dr. Ayre proceeds to illustrate the pathology of marasmus by facts connected with the functions of the alimentary canal. The different parts of the digestive apparatus have an intimate connexion with each other, by continuity of surface and by nervous intercourse; their severally subservient actions are excited by sympathy, and by the successive application of transmitted stimulant materials. Amongst organs so reciprocally dependent, and so consentaneous in their actions, disorder, in whichever originating, is easily communicated to others of the same system: thus, derangement of the stomach will produce irregularity in the action of the liver; and this latter disorder in the functions of the duodenum, whence further disturbance is propagated. Thus it seems hardly possible that disorder should occur, however originating, in any part of this system, without the associate organs participating more or less in the morbid affection; and by these means the process dependent on their joint offices is imperfectly performed. It is difficult to determine which of the chylopoïetic viscera is, in the disease under consideration, most commonly the first to suffer disorder; but we are enabled to detect the existence of derangement in several of the more important, by an obvious deviation from their accustomed actions. Thus, we observe the stomach craving or loathing food, rejecting the ingesta, or throwing out viscid phlegm or other vitiated secretions. Of deranged hepatic function we have tolerably accurate means of judging, by the appearances of the alvine excretions: in these we observe an excess, a suspension, a deficiency, or an unhealthy state, in the secretion of the liver. These are unequivocal indications of the seat of disorder; and these are commonly present, more or less, in the disease in question. In marasmus, Dr. Ayre thinks that disorder of the liver is rarely absent. In speaking of the derangements of function of this viscus, he is of opinion that the dark matter which is frequently voided by stool owes its colour to the presence of blood, and that this is effused from the extremities of the minute branches of the vena portarum, in consequence of congestion in this system of vessels. The proof of this state of congestion he draws from various considerations, and he believes it to exist in both the acute and chronic stages of marasmus. It is caused, he says, by suspension of the secretory function of the liver, and is relieved in several ways,—by a restoration of the secretion of bile, which, when in excess, constitutes cholera morbus,—or by an effusion from the secreting extremities of the veins of unchanged blood. To this, the author thinks, is to be attributed the black colour of the motions. This effusion he considers to take place sometimes in large quantity, and, when carried off by the bowels, it constitutes melæna; when it is thrown up by the convulsive action of the stomach, it becomes the disease hæmatemesis. The customary means of relief in these affections coincide with the view which is here given of their origin. Medicines which clear out the contents of the bowels, and promote the healthy function of the liver, relieve the congestive state of its venous system. This opinion of Dr. Ayre as to congestion in the hepatic vessels, is extended to its almost necessary occurrence in the early stage of disorder, wherein the natural action of these vessels is suspended; and he considers it to be almost invariably present as a precursory symptom to more obvious biliary derangement. This forms the main feature in his view of bilious disorders, and gives the bias to his practice; and, perhaps, it would not be easy to refute the author's doctrine. His view of the pathology of marasmus is recapitulated in the following passage :—

"1st. That this disorder consists in a deranged and imperfect action in the secretory function of the liver, and a consequent deficient and unhealthy secretion of bile, as is manifested by the alvine discharges not having that colour which is always imparted to them by it, when it is secreted in a healthy state and in the proper quantity.

"2d. That this derangement in the functions of the liver commonly arises from a disorder commencing in the stomach; for the function of digestion is performed by organs whose actions, by means of a nervous union established among them for this purpose, are rendered accordant and co-operative; the healthful action of the liver depending upon a stimulus imparted to it by the stomach in obedience to this law.

"3d. That in certain deranged states, therefore, of the stomach, the precise nature of which is unknown, there is either a morbid or an imperfect stimulus given to the liver, by which its secretory function is impeded, and a bilious fluid produced that is deficient in its quantity, and commonly of a morbid kind.

"4th. That, as an interruption in the accustomed actions of a secreting organ occasion a congestion of its vessels, the diminished secretion of the bile gives rise to a congestive state of the vena portarum and its branches; and, in some cases, to a similar state in those organs whose venous system is associated with that of the liver.

"5th. That, in consequence of those efforts which nature makes to free herself from disorder, this congestive state is sometimes spontaneously removed by a copious secretion of bile, constituting the bilious diarrhœa or the cholera morbus; and that, in other cases, it is temporarily relieved by an hæmorrhoidal flux, or by the discharge of blood from the loaded extremities of the vena portarum; occasioning, in this latter case, and when in small quantities, the black and tar-like, and often putrid and fetid, stools; and, when in excess, the idiopathic hæmatemesis or melæna.

"6th. That, whilst this congestive state of the liver produces an assemblage of symptoms resembling in many points the acute inflammation of that organ, it differs essentially from that state in many important particulars. For, in the acute inflammation of the liver, it is the arterial action of the organ that is excited, and the congestion (if the expression be allowable) is arterial; the secretory function of the organ, from its being carried on by a distinct class of vessels, partaking only secondarily and partially in its effects; whilst, in the venous congestion of the liver, consequent upon an interruption in its secretory action, the arterial system of the liver is necessarily but little, if at all, affected, the congestive state in that organ being, in all probability, limited to the vena portarum and its branches.

"7th, and lastly. That the indications for the removal of these morbid but dissimilar states will, therefore, necessarily be different. The inflammation in the liver will demand the same treatment which is applicable to inflammation in other parts of the body; for it differs in nothing from that state in them, either in its origin or nature: whereas, in the other disorder, from its having nothing in common with inflammation, it will not, as I have repeatedly found, be benefited by venæsection or by blistering, and the severe and antiphlogistic regimen; but the principal object to be attained will consist in a renewal of the healthy secretory action of the liver, as it is from the interruption of this, that the congestive state, with its immediate train of painful symptoms, has arisen."

The remote causes which Dr. Ayre assigns to marasmus are cold—irregularities of diet—excess in the use of spirits—the impure air of crowded or close situations—certain eruptive fevers—sedentary employments, &c. &c. On each of these he specially dilates; and, in the part devoted to the consideration of irregularities of diet, are introduced some highly valuable observations on the dietetic treatment of infancy, which, in the hands of ignorance and depravity, is so commonly productive of disease and death. The very flagrant violation of the plainest dictates of nature, committed in the mismanagement of infants, almost from the moment of their birth, amongst the uninformed, must be familiar to the notice of every practitioner in the crowded haunts of man. These abuses the author takes an opportunity of exposing, and of justly reprobating; and we cannot but regret that his remarks on this subject should not have a wider range than the circle of professional readers will afford to them.

In the treatment of marasmus, Dr. Ayre lays down these general indications of cure:—1st, to correct the disordered action of the liver, and remove the congestive state of that organ; 2nd, to cleanse the bowels of their morbid secretions, and the imperfectly-digested matters collected there; and, 3d, to lessen or avoid all those causes which tend to aggravate the complaint.

The two former of these indications call, of course, for the exhibition of medicines of the purgative class: but, of the agents of this order, he considers none so immediately adapted to fulfil the first intention as calomel, and that given in very minute doses. The doses, indeed, used by Dr. Ayre are so small as almost to appear inadequate to the design; but against the evidence of facts it is vain to reason, and the author says he finds his practice almost invariably successful. To the submuriate of mercury, given in such quantities, the author ascribes, not only a power of exciting the biliary secretion when suspended, but of reducing it to a healthy proportion when in excess. Thus, he finds it equally serviceable in bilious diarrhœa or cholera morbus, as in those states of disorder where the bile is either not poured out or secreted in sufficient quantity. Keeping these properties in view, this medicine is not given in doses to excite purging, but is made to precede, in very minute and divided quantities, the exhibition of medicines for the avowed purpose of clearing out the bowels. Dr. Ayre cautiously avoids giving the calomel so as to affect the mouth, diminishing the quantity or frequency of the doses, and keeping an open state of the bowels by aperients. Of the last-mentioned class of medicines, a preference is given to the neutral salts, as, in evacuating the contents of the intestines, they at the same time increase the secretions of their surface. But these purgatives are not exclusively used, as the author says it is often useful to act upon the different portions of the intestinal tube. Purging is not considered expedient in marasmus; but, when the morbid accumulation of irritating matter has been dislodged, a regular state of evacuation must be maintained. An emetic in the commencement of the attack is often serviceable; in the later stages it is rarely useful. But we cannot follow the author through the whole of his curative views: suffice it to say, that they are characterized by sound judgment, raised on principles derived from rational deduction, and confirmed by the convictive test of experience.

